# Neonatal Nutrition Predicts Energy Balance in Young Adults Born Preterm at Very Low Birth Weight

**DOI:** 10.3390/nu9121282

**Published:** 2017-11-24

**Authors:** Hanna-Maria Matinolli, Petteri Hovi, Esko Levälahti, Nina Kaseva, Patricia P. Silveira, Katri Hemiö, Anna-Liisa Järvenpää, Johan G. Eriksson, Sture Andersson, Jaana Lindström, Satu Männistö, Eero Kajantie

**Affiliations:** 1 Department of Public Health Solutions, National Institute for Health and Welfare, FI-00271 Helsinki, Finland; petteri.hovi@thl.fi (P.H.); esko.levalahti@thl.fi (E.L.); nina.kaseva@fimnet.fi (N.K.); katri.hemio@thl.fi (K.H.); jaana.lindstrom@thl.fi (J.L.); satu.mannisto@thl.fi (S.M.); eero.kajantie@thl.fi (E.K.); 2 Institute for Health Sciences, University of Oulu, FI-90014 Oulu, Finland; 3 Children’s Hospital, University of Helsinki and Helsinki University Hospital, FI-00290 Helsinki, Finland; al.jarvenpaa@fimnet.fi (A.-L.J.); sture.andersson@hus.fi (S.A.); 4 Ludmer Centre for Neuroinformatics and Mental Health, Douglas Mental Health University Institute, McGill University, Montreal, QC H3T 1E2, Canada; patricia.silveira@mcgill.ca; 5 Department of General Practice and Primary Health Care, University of Helsinki and Helsinki University Hospital, FI-00014 Helsinki, Finland; johan.eriksson@helsinki.fi; 6 Folkhälsan Research Center, FI-00280 Helsinki, Finland; 7 PEDEGO Research Unit, MRC Oulu, Oulu University Hospital and University of Oulu, FI-90014 Oulu, Finland

**Keywords:** neonatal nutrition, programming, very low birth weight, preterm, premature, food intake, energy intake, energy expenditure, exercise

## Abstract

Epidemiological studies and animal models suggest that early postnatal nutrition and growth can influence adult health. However, few human studies have objective recordings of early nutrient intake. We studied whether nutrient intake and growth during the first 9 weeks after preterm birth with very low birth weight (VLBW, <1500 g) predict total energy intake, resting energy expenditure (REE), physical activity and food preferences in young adulthood. We collected daily nutritional intakes and weights during the initial hospital stay from hospital records for 127 unimpaired VLBW participants. At an average age 22.5 years, they completed a three-day food record and a physical activity questionnaire and underwent measurements of body composition (dual X-ray absorptiometry; *n* = 115 with adequate data) and REE (*n* = 92 with adequate data). We used linear regression and path analysis to investigate associations between neonatal nutrient intake and adult outcomes. Higher energy, protein and fat intakes during the first three weeks of life predicted lower relative (=per unit lean body mass) energy intake and relative REE in adulthood, independent of other pre- and neonatal factors. In path analysis, total effects of early nutrition and growth on relative energy intake were mostly explained by direct effects of early life nutrition. A path mediated by early growth reached statistical significance only for protein intake. There were no associations of neonatal intakes with physical activity or food preferences in adulthood. As a conclusion, higher intake of energy and nutrients during first three weeks of life of VLBW infants predicts energy balance after 20 years. This association is partly mediated through postnatal growth.

## 1. Introduction

Obesity is a consequence of a chronic imbalance between energy intake and energy expenditure [1,2] but in humans there is considerable individual variation in energy balance, preference to energy-rich, palatable foods and susceptibility to weight gain as a result of overeating [3,4]. Epidemiological studies as well as intervention studies on animals have proposed that energy balance is partly programmed already during fetal life or infancy (for a review, see [5]). According to the Developmental Origins of Health and Disease (DOHaD) theory, developmental programming occurs when a fetus or infant reacts to the early life environment making predictive adaptations and consequent metabolic adjustments that are sustained in later life [6]. These adaptations may lead to increased risk for developing a number of chronic diseases in later life [7]. 

Consistent with the DOHaD theory, there is accumulating epidemiological evidence suggesting that preterm birth (<37 weeks of gestation) especially at very low birth weight (VLBW, <1500 g), by exposing an individual to an adverse early life environment, may result in higher risk for cardiometabolic disease [8,9,10,11,12]. Epidemiological data mostly on subjects who were born small for gestational age (SGA) have shown that metabolic abnormalities in adulthood are associated with catch up growth during early life, which also preterm born subjects often experience [13,14,15,16]. Animal studies [17,18] as well as epidemiological and clinical studies have suggested that early nutritional intake, human examples of which include shorter duration of breastfeeding and higher protein content in infant formula, is associated with cardiometabolic risk factors in later life [19,20,21]. Further, early life environment, including maternal diet during pregnancy [22] and restricted fetal growth [23,24], have been associated with specific food preferences in adult age. A limitation of most human studies is that they have relied on proxies, as direct assessment of early nutrition has not been available. The few exceptions include trials assessing the long term effects of protein content in infant formula [25] or observational studies in those born preterm at VLBW whose daily nutrient intake during the weeks to months long neonatal hospital stay is recorded in detail [26,27]. However, most of these studies have not yet extended to adult age. 

We now extend past studies by investigating whether nutrition during the early weeks after preterm birth at VLBW is associated with energy balance and food preferences in young adult age and whether the possible associations are mediated through growth during the same period. We have previously shown [28] that higher protein intake during the first three weeks after birth at VLBW predicts higher lean body mass and lower resting energy expenditure per unit lean body mass. Based on the above, we hypothesize that lower early intakes of energy and protein are associated with higher intake of energy and nutrients, lower resting energy expenditure and energy expenditure from physical activity and preference for palatable foods in young adult age and that these associations are mediated through growth during the same period.

## 2. Materials and Methods

The participants came from the Helsinki Study of Very Low Birth Weight Adults [8]. The source cohort comprises 335 subjects born preterm at VLBW between 1978 and 1985 and discharged alive from the neonatal intensive care unit of Children’s Hospital at Helsinki University Central Hospital. When reaching young adulthood in 2004–2005, the VLBW and control participants residing in greater Helsinki area were invited to participate in a clinical follow-up examination: of the 255 VLBW subjects invited, 166 (65.1%) (mean age 22.5 years) participated. All participants gave their written informed consent. The study was approved by the Ethics Committee of Children and Adolescents Diseases and Psychiatry at the Helsinki University Central Hospital (333/E0/2003). While the cohort also includes controls born at term [8], they are not included in the current study, which focuses on early nutrition after preterm birth.

### 2.1. Data Collection

As described in detail [28], we collected the daily nutritional intake during the initial hospital stay from hospital records for 158 VLBW participants. From the data collected, we calculated the mean intakes of energy, protein, fat and carbohydrate per kg body weight per day for the first nine weeks of life [28]. The nine-week data were further divided into three three-week periods and the mean intake of energy and nutrients during the periods of Weeks 1–3, Weeks 4–6 and Weeks 7–9 serve as our main exposure variables [28]. After exclusion of subjects with incomplete hospital records (*n* = 17) and subjects with neurosensory impairments (*n* = 14), we ended up with data for 127 VLBW participants.

Weight measurements came from the hospital records. Daily weight measurements were interpolated from measurements around the seven consecutive days. The absolute measurements were transformed into standard deviation (SD) units according to Finnish growth standards [29] and used in the analyses. 

Total energy intake per day in adult age was estimated based on the total energy intake reported in a three-day food record [30,31], which 155 VLBW participants completed prior to the clinical examination. A trained study nurse instructed the participants to report everything they ate and drank at the times foods are eaten, during a three-day period. The participants were given a picture booklet of typical portion sizes of foods in order to help in estimating the weight of foods eaten [32]. Mean daily consumption of foods and intakes of energy and macronutrients were calculated by using a dietary analysis program based on the national FINELI database [33]. We report total energy intake in units of kilocalories per day (kcal/day) and intake of macronutrients as percentage of total energy intake/day (E%). The distributions for variables describing food intakes per day were non-normal so we used log-transformed values in analyses and therefore present the results as back-transformed values. As previously reported, body composition was measured by whole-body dual X-ray absorptiometry (DXA) (software version 12.3:3, Hologic, Bedford, MA, USA) [34] (*n* = 118) and resting energy expenditure (REE) at rest by indirect calorimetry (Deltatrac II; Datex, Helsinki, Finland) when the device was available (*n* = 96). Based on these measurements, we calculated the ratio of REE/lean body mass (LBM) (kg) (relative REE) [35]. In path analysis, we used the relation of total energy intake to LBM (relative energy intake) to assess the energy intake per kg of LBM, similar to relative REE. Physical activity level was calculated in metabolic equivalent hours (MET) per week based on a self-report from questionnaire on: (1) light (assuming a value of 3 MET); (2) moderate to vigorous (5 MET); and (3) commuting physical activity (4 MET) [36]. One hundred fifteen participants with no missing information on early nutrition, DXA measurements and food record data were included in the analyses. 

Several sociodemographic, medical, and behavioral characteristics were assessed as possible confounders. Pre- and neonatal data were extracted from standardized structured medical records routinely kept at birth hospitals. Gestational age of the participants was based on last menstrual period and confirmed after birth by Dubowitz examination by one neonatologist (ALJ). Birthweight SD score was calculated based on Finnish standards [29] and preeclampsia was diagnosed using standard criteria [37]. Bronchopulmonary dysplasia (BPD) was assessed by one neonatologist (ALJ) based on the Northway criteria [38] and septicemia was diagnosed if the infant had symptoms and if the blood culture was positive. The diagnosis of persistent ductus arteriosus (PDA) came from hospital records. Maternal smoking during pregnancy (yes/no) was also collected from medical records. Information regarding parental education was obtained from questionnaires and was used as an indicator of socioeconomic status (categorized into four levels). Smoking habits (daily smoking, yes/no) and habitation (living at parental home, yes/no) were also obtained from questionnaire. Weight and height were measured by trained personnel and body mass index (BMI) was calculated as kg/m^2^.

### 2.2. Analyses

**Figure 1:**
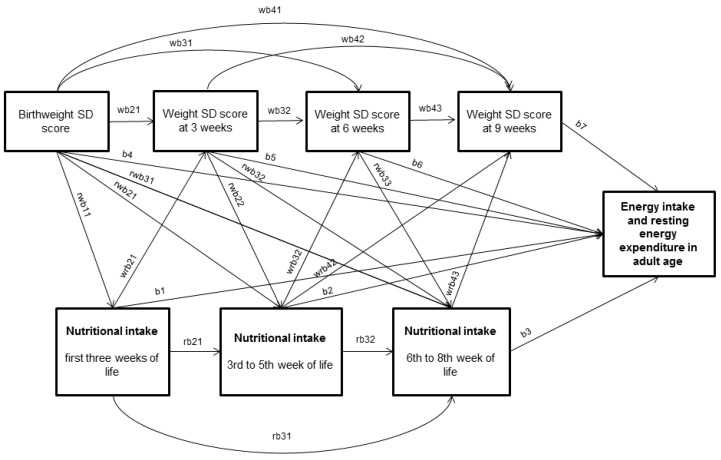
Hypothetical full path model for neonatal nutrient intake with three time points, body weight with four time points and relative energy intake in adult age. Paths are labeled using following abbreviations: wb* for paths between weight SD scores, rb* for paths between nutritional intake variables, rwb* for paths from weight SD scores to nutritional intake variables, wrb* for paths from nutritional intake variables to weight SD scores and b* for paths from weight SD scores and nutritional intake variables to adult age outcome. For wb and rwb paths, “*” represents the order of variables from left to right of both dependent and explanatory variable.

We used linear regression analysis to examine the relationships of early nutrition and early growth with the main outcome variables, relative energy intake, relative REE, physical activity (MET) and the intake of macronutrients and foods. For two of the outcomes, REE and energy intake, we used path analysis [39] (described in more detail in Appendix A) to further investigate the associations we had found by multiple regression analyses between neonatal nutrition and growth and adult outcomes (*p* < 0.10 in univariate regression model). All path models were based on general hypothetical full path model shown in Figure 1 with the hypothesis that neonatal nutrition (total energy, protein, fat or carbohydrate intake) explains the levels of outcomes (relative energy intake or expenditure) and it may be partly mediated through neonatal growth during the same time period. The model uses all time points available for neonatal variables. All available data that had non-missing values on dependent variables were included. 

All path models were estimated using Mplus (version 5.1, Muthén & Muthén, Los Angeles, CA, USA) [40]. Regression analyses and descriptive statistics were estimated using Stata (SE, version 14.1, StataCorp LLC, College Station, TX, USA) [41]. All path models, except for multiple imputation sensitivity analyses, were estimated using missing data handling techniques with maximum likelihood on all available data. Alternative nested sub models were tested using χ^2^-difference tests calculated as −2 times the difference in log-likelihood values of the alternative model with more estimated parameters and simpler model with some parameters fixed, and degrees of freedom as difference of freely estimated model parameters in these alternative models. Bias corrected confidence intervals (CI) were estimated with 1000 bootstrap draws. 

## 3. Results

The peri- and neonatal characteristics of the study group are presented in Table 1. The mean energy and nutrient intakes during the early weeks were lower than those currently recommended [28,42]. We observed no statistically significant interaction between sex and early nutrient intake in further analyses so we analyzed the data for both sexes combined.

### 3.1. Regression Analysis

Results from the regression analyses are presented in Table 2. In the initial regression analyses 10 kcal/kg/day higher energy intake during the first three weeks of life was associated with 1.3 kcal (95% CI 0.06 to 2.62) lower total energy intake per 1 kg of LBM in adult age. A 1 g/kg/day higher early protein (−5.12 kcal (−10.72 to 0.49)) or fat (−1.75 kcal (−3.55 to 0.05)) intake tended to be associated with lower relative energy intake. Early nutritional intakes were associated with relative REE in adult age (total energy *p* = 0.02, protein energy *p* = 0.006 and fat energy *p* = 0.04). Weight measurements during the first nine weeks of life were statistically significantly associated with relative REE (weight SD score at three weeks energy *p* = 0.04, weight at six weeks energy *p* = 0.01 and weight at nine weeks energy *p* = 0.001), and weight SD score at nine weeks of age was associated with relative energy intake (energy *p* = 0.04).

When self-reported physical activity and intake of food items were assessed as outcomes, we found no statistically significant associations with early life exposures assessed in this study. Adjustments for potential early life confounders or current mediating characteristics did not change the results (Table S1).

### 3.2. Path Analysis

We used path analysis to further investigate the total, direct and indirect effects between neonatal nutrition and growth and adult outcomes. In these analyses, we focused on relative energy intake and relative REE because these outcomes had been predicted by early nutrition and growth in the multiple regression analyses. The path analysis was based on the hypothetical path diagram shown in Figure 1. 

Path coefficients and 95% bias corrected CI show that energy intake during the first three weeks of life predicted both relative total energy intake and relative REE. The adjustments for potential early life confounders did not change the results, except adjustment for neonatal factors strengthened the association with total relative energy intake (Table 3). Protein and fat intakes during the first three weeks of life were associated with relative REE in all models and also with relative total energy intake when fully adjusted for confounding factors (Model 4). 

**Figure 2:**
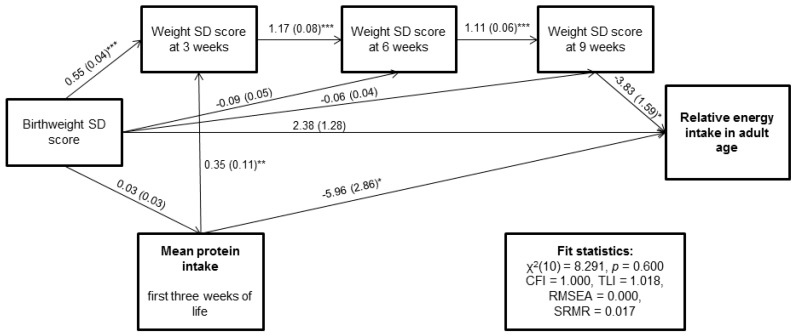
Graphical representation of path model estimated for protein intake during the first three weeks of life, body weight with four time points and relative energy intake in adult age (*n* = 109). Paths represent path coefficient and standard error in parenthesis. Significance of estimated path is noted as * *p* < 0.05, ** *p* < 0.01 and *** *p* < 0.001. Abbreviations used: CFI Comparative Fit Index; RMSEA Root Mean Square Error of Approximation; SD Standard Deviation; SRMR Strandardized Root Mean Residual; TLI The Tucker-Lewis Index.

Table 4 shows the test results from five alternative nested models with full adjustments. The full model comprises all other variables included in the hypothetical model, excluding nutrition during Weeks 4–6 and Weeks 7–9 of life. Based on Table 4 testing nested Models 1 to 3, hypotheses on relationships between neonatal variables are accepted. Figure 2 and Figures S1 and S2 show the estimation results and fit results for subModel 3 for relative energy intake. Fit statistics indicate adequate fit for all three models. The test on nested Model 4 (Table 4) shows that there may be indirect effects involved for neonatal protein and fat intakes on relative energy intake in adult age. The test for the effect of neonatal energy intake on relative energy intake shows borderline significance for indirect effect. Regarding relative REE, indirect effects were not found (*p* for Model 4 > 0.05). Tests regarding the nested Model 5 show significant *p* values, as expected based on the results from earlier regression and path analyses (Table 3 and Table 4). 

Table 5 shows the model derived direct, indirect and total effect estimates with 95% bias corrected CI for the association between early nutrition and relative energy intake in adult age. The total effects were mostly explained by the direct effects from the early nutrition. A path mediated by early growth was present but reached statistical significance only with protein intake. 

### 3.3. Sensitivity Analyses

We reran the analyses of early nutrient intakes by adjusting for the age at discharge from hospital. We also performed the multiple imputations. The point estimates for the indirect effects attenuated slightly, but remained significant for energy, protein and became so for fat intake (Table S2).

## 4. Discussion

In this cohort study with a 20+ years follow-up, we found that higher intake of energy, protein and fat during the first three weeks after birth of a VLBW infant predicted lower relative energy intake and lower relative REE in young adult life. However, early nutrition was unrelated to energy expenditure from physical activity or to food preferences as assessed by consumption of food items. We have previously shown that higher early nutrition during the first weeks of life is associated with higher LBM and REE and lower REE per unit LBM in the same population of young adults born with VLBW [28]. We now extend these findings by showing that the excess LBM associated with more adequate early nutrition is on average metabolically less active, as indicated by reduced energy intake in addition to REE, and that these associations are in part but not solely mediated through early growth. It is of note that the mean intakes of nutrients in our study were low according to the current nutritional guidelines and accompanied by a much slower growth than would have been expected in utero or in VLBW infants in adequate care today.

Most human studies assessing the effects of infant and child nutrition on adult adipogenic factors have used growth as a proxy for nutrient intake [43,44]. Among healthy term infants, the direction or relationship with infant growth varies: in less affluent populations, the risk factors are predicted by slower infant growth [43,45], whereas, in contemporary affluent populations, cardiometabolic risk factors are usually predicted by more rapid infant growth [46]. This resembles the situation in SGA infants, who are growth restricted already during fetal life and often experience catch-up growth in the neonatal period. Among these infants faster catch-up growth is suggested to be predictive of higher levels of cardiometabolic risk factors [13,47]. Few studies have assessed this in VLBW infants; in the present cohort, more rapid growth between birth and term predicted insulin resistance at age 20+, although this was restricted to VLBW individuals born SGA [8]. It is of note that in VLBW infants more rapid growth between birth and term also predicts favorable outcomes such as better cognitive functioning [48] and social interaction [49].

Direct evidence on the long term effects of the early postnatal nutrition is provided by trials on protein content in infant formulas showing in individuals born SGA that those receiving higher protein levels have higher fat mass at 5–8 years of age [25]. A corresponding trial in infants born at term suggests higher rates of obesity as evaluated by BMI at age six years [21]. Another source of direct evidence are observational studies in children and adults born preterm at VLBW whose daily nutrient intake during the weeks to months long neonatal hospital stay is recorded in detail. However, previous studies using such records are rare [26,27]. To our knowledge, the present study is the first to take into account both early nutrition and early growth in predicting adult outcomes in preterm infants.

Our path model showed a direct effect of 5.9 kcal/kg/day lower adult caloric intake when the early protein intake was increased by 1 g/kg/day. This corresponds to an on average 380 kcal, or over 0.7 SD, lower daily energy intake in young adult age in an individual weighing 65 kg. Total relative energy intake should in this context be seen as a proxy of total energy expenditure in particular as most study participants were not overweight or obese. Most of total energy expenditure is constituted by REE, which, accordingly, we found to be associated with early nutrient intake. In other words, VLBW infants who were undernourished during the first three weeks of life have metabolically more active tissue in young adult age. Other components of total energy expenditure include physical activity and thermic effect of food. We found no association with energy expenditure from physical activity which, however, should be treated with caution as the proportion of physical activity of total energy expenditure is low, in our study approximately 20%. We did not measure the thermic effect of food, which depends on diet but generally constitutes not more than ~10% of total energy expenditure

One consistent finding throughout the experimental animal studies on programming of food and nutrient intake is that malnourishment during the early stages of life is associated with the intake of more palatable and more energy-dense foods in adult age; in humans, this has been shown in the Dutch Famine study [22], and this was also our hypothesis. Studies comparing preterm and term-born adults have shown less affinity for protein-rich foods and higher affinity for sweets [50] and lower intake of fruits and vegetables [32], broadly consistent with this. Moreover, a study in 56–70-year-olds of the Helsinki Birth Cohort study showed lower birth weight to be associated with a higher fat intake [23]. However, we were not able to see any association between early nutrition and intakes of food items or macronutrients in adult age, suggesting that the associations we found between neonatal nutrient intake and adult energy balance are unlikely to be mediated through specific food preferences. This is consistent with a Jamaican study that compared adult food preferences in an experimental free-choice situation between adult survivors of two types of childhood malnutrition: marasmus (non-edematous) and kwashiorkor (edematous). Of these, marasmus is more common among children born with lower birth weights and children with marasmus maintain their metabolic integrity during malnutrition. However, in adulthood, there was no difference in macronutrient intakes between the groups [51]. These reports of a lack of association, however, should be interpreted with caution as our study and the study in malnutrition survivors assessed food and nutrient intakes rather than preferences per se. 

Specific biologic mechanisms underlying the associations between early nutrition and energy balance in adulthood remain to be elucidated. However, animal studies on early life factors and leptin levels in later life have suggested that high-fat diet during gestation and lactation as well as restricted diet during pregnancy results in impaired leptin sensitivity in offspring [52]. Leptin, a cytokine produced by the adipose tissue, acts at different levels of the Central Nervous System and regulates energy intake and expenditure. Interestingly, leptin (as well as insulin) modulates the activity of mesocorticolimbic dopamine neurons [53], and dopamine function also is modified by early life nutrition [54,55]. As variation in dopamine function contributes to obesity through alterations in energy expenditure and activity [56], changes in this system via differences in leptin modulation can potentially be involved in the current findings.

Strengths of the present study include the unique, detailed data on early nutrition and growth during the first weeks of life and the path analysis method applied. We were also able to access extensive data on potential confounders, most importantly neonatal illnesses and maternal pregnancy disorders. Several limitations however exist. One of the major limitations is the small sample size. Possible selection bias has been discussed previously in [28]. For assessing the adult age outcomes, we used valid, exact measures in measuring body composition and REE. Physical activity was assessed by a relatively simple questionnaire, which may introduce inaccuracy. To assess the average daily energy intake, we used self-reported data from a three-day food record thus energy intake may be under-reported and accuracy may be an issue. However, the participants used a picture booklet for assessing accurate portion sizes, and the food diaries were checked by nutritionist. Therefore, we believe that food and nutrient intakes are reliable [57]. In addition, correlation between the self-reported total energy intake and REE was 0.43 (*p* < 0.001) showing good reliability. Food diaries are designed to assess food intake in general rather than food preferences, which were under our hypothesis. We were not able to differentiate enteral and parenteral feeding in our analyses which could have played a role in programming food preferences. In addition, we observed the naturally occurring variation in feeding that was rather low at the time points assessed. Finally, due to lacking data we were unable to take in account eating behaviors, growth or lifestyle factors in late infancy, childhood or adolescence in our analyses, which potentially affect the outcomes assessed in our study as well.

## 5. Conclusions

At rather low neonatal energy and nutrient intakes, higher intake was independently associated with lower relative energy intake as well as lower relative REE in adult age. A part of this association was mediated by early growth; however, the total effect was mainly driven by the direct effect from early nutrition. Our study reinforces suggestions that adult energy balance is partly programmed by relatively small variations in neonatal nutrition and growth.

## Figures and Tables

**Table 1:** Characteristics of the study participants.

Characteristic		*n* (%)/Mean ± SD/Mean (25th, 75th Percentiles)	*n*
Sex, women		68 (59.1%)	115
**BIRTH CHARACTERISTICS**			
Birth weight, g		1113 ± 217	115
Gestational age, week		29.0 ± 2.1	115
Birth weight SD score		−1.17 ± 1.53	115
Maternal smoking during pregnancy		22 (19.1)	115
Maternal pre-eclampsia		21 (18.3)	115
**NEONATAL CHARACTERISTICS**			
Neonatal sepsis		8 (7.1)	112
Bronchopulmonary dysplasia		22 (19.1)	115
Indomethasin treatment or PDA operation		33 (29.2)	113
Mechanical ventilation, days		12.5 (0,17)	113
**NEONATAL NUTRITION AND GROWTH**			
Weight SD score at 3 weeks		−3.22 ± 0.86	111
Weight SD score at 6 weeks		−3.42 ± 0.85	103
Weight SD score at 9 weeks		−3.37 ± 0.87	74
Mean energy intake 1st to 3rd weeks, kcal/kg/day		90.4 ± 10.6	115
Mean energy intake 4th to 6th weeks, kcal/kg/day		118.8 ± 15.0	112
Mean energy intake 7th to 9th weeks, kcal/kg/day		124 ± 13	102
Mean protein intake 1st to 3rd weeks, g/kg/day		1.4 ± 0.4	115
Mean protein intake 4th to 6th weeks, g/kg/day		1.9 ± 0.4	112
Mean protein intake 7th to 9th weeks, g/kg/day		2.1 ± 0.5	102
Mean fat intake 1st to 3rd weeks, g/kg/day		4.3 ± 1.1	115
Mean fat intake 4th to 6th weeks, g/kg/day		5.9 ± 1.0	112
Mean fat intake 7th to 9th weeks, g/kg/day		6.1 ± 0.9	102
Mean carbohydrate intake 1st to 3rd weeks, g/kg/day		11.1 ± 1.3	115
Mean carbohydrate intake 4th to 6th weeks, g/kg/day		12.4 ± 1.3	112
Mean carbohydrate intake 7th to 9th weeks, g/kg/day		12.9 ± 1.3	102
Term weight SD score		−2.93 ± 0.93	104
**CURRENT CHARACTERISTICS**			
Highest parental education			
	Elementary	11 (9.7)	
	High School	29 (25.7)	
	Intermediate	41 (36.3)	
	University	32 (28.3)	
Age at clinical examination, year		22.5 (2.1)	
Daily smoking		29 (25.7)	
Height at clinical examination, cm	Women	163.1 (7.1)	68
	Men	174.9 (7.4)	47
Weight at clinical examination, kg	Women	59.1 (12.0)	68
	Men	65.9 (11.7)	47
BMI, kg/m^2^	Women	22.2 (3.7)	68
	Men	21.5 (3.1)	47
Lean body mass, kg	Women	39.9 (5.5)	68
	Men	53.3 (7.7)	47
Resting energy expenditure, kcal/24 h	Women	1464 (214)	56
	Men	1817 (218)	36
Total caloric intake, kcal	Women	1597 (447)	67
	Men	2170 (555)	42
Protein, E%	Women	15.9 (4.0)	67
	Men	16.2 (4.1)	42
Fat, E%	Women	33.6 (7.0)	67
	Men	37.3 (6.9)	42
Carbohydrate, E%	Women	47.9 (7.3)	67
	Men	42.7 (6.6)	42

**Table 2:** Results from the univariate regression analysis for the complete set of predictors on the outcomes.

Predictors	Outcomes					
	Relative Energy Intake (*n* = 84–107)		Relative REE (*n* = 55–90)		Physical Activity, MET Hours (*n* = 78–122)	
	Unstandardized Regression Coefficient (95% CI)	*p*	Unstandardized Regression Coefficient (95% CI)	*p*	Unstandardized Regression Coefficient (95% CI)	*p*
Energy expenditure, 10 kcal/24 h	−0.06 (−0.15 to 0.02)	0.19	−0.02 (−0.05 to 0.01)	0.10	0.12 (−0.00 to 0.24)	0.29
Birth weight SD score	−0.64 (−2.01 to 0.74)	0.36	−0.4 (−0.88 to 0.08)	0.12	−0.75 (−2.63 to 1.14)	0.50
Mean energy intake 1st to 3rd weeks, kcal	−1.34 (−2.62 to −0.06)	0.04	−0.51 (−0.96 to −0.05)	0.02	0.67 (−1.16 to 2.51)	0.96
Mean protein intake 1st to 3rd weeks, g	−5.12 (−10.72 to 0.49)	0.07	−2.44 (−4.40 to −0.49)	0.006	3.13 (−4.74 to 11.0)	0.67
Mean fat intake 1st to 3rd weeks, g	−1.75 (−3.55 to 0.05)	0.06	−0.67 (−1.33 to −0.02)	0.04	0.97 (−1.61 to 3.56)	0.99
Mean carbohydrate intake 1st to 3rd weeks, g	−0.82 (−2.41 to 0.78)	0.31	−0.23 (−0.77 to 0.32)	0.37	0.93 (−1.25 to 3.12)	0.34
Mean energy intake 4th to 6th weeks, kcal	−0.26 (−1.67 to 1.15)	0.72	−0.27 (−0.77 to 0.23)	0.24	−0.48 (−2.41 to 1.44)	0.56
Mean protein intake 4th to 6th weeks, g	−3.77 (−8.81 to 1.26)	0.14	−0.91 (−2.58 to 0.75)	0.18	−1.27 (−7.80 to 5.27)	0.87
Mean fat intake 4th to 6th weeks, g	−0.67 (−2.79 to 1.45)	0.53	−0.18 (−0.93 to 0.57)	0.59	−1.07 (−3.92 to 1.78)	0.40
Mean carbohydrate intake 4th to 6th weeks, g	0.71 (−0.89 to 2.32)	0.38	−0.11 (−0.70 to 0.48)	0.65	−0.32 (−2.50 to 1.86)	0.93
Mean energy intake 7th to 9th weeks, kcal	0.65 (−1.01 to 2.31)	0.44	0.08 (−0.48 to 0.64)	0.71	−0.48 (−2.80 to 1.83)	0.64
Mean protein intake 7th to 9th weeks, g	−2.91 (−7.48 to 1.65)	0.21	−0.94 (−2.51 to 0.64)	0.15	2.11 (−4.11 to 8.33)	0.72
Mean fat intake 7th to 9th weeks, g	0.48 (−1.89 to 2.84)	0.69	0.4 (−0.39 to 1.19)	0.31	−1.23 (−4.49 to 2.04)	0.63
Mean carbohydrate intake 7th to 9th weeks, g	0.9 (−0.87 to 2.68)	0.31	−0.06 (−0.64 to 0.53)	0.98	0.17 (−2.19 to 2.53)	0.73
Weight SD score at 3 weeks	−1.74 (−4.28 to 0.80)	0.18	−0.89 (−1.78 to 0.00)	0.04	−1.03 (−4.39 to 2.33)	0.56
Weight SD score at 6 weeks	−1.26 (−3.97 to 1.46)	0.36	−1.01 (−1.91 to −0.12)	0.01	−0.18 (−3.80 to 3.43)	0.99
Weight SD score at 9 weeks	−3.27 (−6.46 to −0.08)	0.04	−1.31 (−2.30 to −0.33)	0.001	−1.03 (−4.64 to 2.57)	0.72
Physical activity, MET/day	−0.07 (−0.21 to 0.07)	0.47	−0.01 (−0.05 to 0.04)	0.94		
Relative MET	−2.67 (−8.6 to 3.26)	0.74	1.55 (−0.51 to 6.61)	0.06		
Relative REE	0.29 (−0.35 to 0.94)	0.41				

**Table 3:** Path coefficients and 95% bias corrected confidence intervals from models including the outcome and one nutrient intake variable (total energy, protein or fat intake) during all three time periods.

Early Nutrition	Relative Energy Intake				Relative Resting Energy Expenditure			
	Model 1 Path Coefficient (95% CI)	Model 2 Path Coefficient (95% CI)	Model 3 Path Coefficient (95% CI)	Model 4 Path Coefficient (95% CI)	Model 1 Path Coefficient (95% CI)	Model 2 Path Coefficient (95% CI)	Model 3 Path Coefficient (95% CI)	Model 4 Path Coefficient (95% CI)
Mean energy intake (10 kcal/kg/day)								
birth to 3rd week	−1.4 (−2.9; −0.1)	−1.6 (−3.1; −0.2)	−1.7 (−3.1; −0.2)	−2.7 (−4.4; −1.0)	−0.50 (−0.94; −0.09)	−0.43 (−0.96; −0.02)	−0.40 (−0.92; −0.00)	−0.41 (−0.91; −0.00)
4th to 6th week	−0.0 (−2.0; 2.0)	−0.2 (−2.3; 1.9)	−0.1 (−2.3; 1.9)	0.1 (−2.1; 2.0)	−0.14 (−0.73; 0.46)	−0.11 (−0.72; 0.48)	−0.17 (−0.77; 0.44)	−0.16 (−0.77; 0.48)
7th to 9th week	0.8 (−1.5; 2.4)	0.6 (−1.5; 2.4)	0.6 (−1.6; 2.4)	0.4 (−1.5; 2.2)	0.06 (−0.49; 0.80)	0.15 (−0.46; 0.87)	0.25 (−0.40; 1.03)	0.27 (−0.43; 1.05)
Mean protein intake (g/kg/day)								
birth to 3rd week	−4.7 (−12.4; 1.5)	−5.7 (−13.4; 1.1)	−5.7 (−13.5; 0.9)	−8.1 (−16.5; −0.3)	−2.81 (−4.73; −0.91)	−2.47 (−4.61; −0.60)	−2.48 (−4.59; −0.66)	−2.53 (−4.80; −0.73)
4th to 6th week	0.4 (−6.6; 8.9)	1.0 (−6.2; 10.2)	0.9 (−6.4; 10.2)	1.6 (−6.1; 11.0)	0.82 (−1.26; 2.83)	0.60 (−1.50; 2.66)	0.62 (−1.61; 2.74)	0.73 (−1.40; 3.00)
7th to 9th week	−2.9 (−8.2; 3.6)	−3.6 (−9.1; 3.0)	−3.5 (−9.1; 3.2)	−2.4 (−8.1; 4.3)	−0.49 (−2.29; 1.42)	−0.09 (−2.07; 1.92)	−0.13 (−2.19; 2.00)	−0.21 (−2.47; 1.99)
Mean fat intake (g/kg/day)								
birth to 3rd week	−1.8 (−4.1; 0.2)	−2.1 (−4.5; 0.0)	−2.1 (−4.5; 0.0)	−3.3 (−6.0; −0.8)	−0.85 (−1.52; −0.21)	−0.74 (−1.48; −0.15)	−0.71 (−1.46; −0.10)	−0.71 (−1.46; −0.08)
4th to 6th week	−0.0 (−2.9; 3.4)	−0.1 (−3.0; 3.4)	−0.0 (−3.0; 3.4)	0.3 (−2.9; 3.9)	0.06 (−0.95; 0.99)	0.09 (−0.92; 1.05)	−0.02 (−1.07; 0.96)	−0.02 (−1.12; 0.95)
7th to 9th week	0.6 (−2.4; 3.3)	0.7 (−2.2; 3.5)	0.6 (−2.2; 3.6)	0.7 (−2.2; 4.0)	0.22 (−0.63; 1.51)	0.25 (−0.73; 1.42)	0.39 (−0.59; 1.67)	0.41 (−0.58; 1.62)

Model 1: Adjusted for birthweight SD score, sex and age at clinical examination; Model 2: Additionally adjusted for gestational age; Model 3: Additionally adjusted for highest parental education, maternal smoking during pregnancy, and maternal preeclampsia; Model 4: Additionally adjusted for neonatal exposures of treatment with ventilator (days), bronchopulmonary dysplasia, septicemia, exchange transfusion, or persistent ductus arteriosus.

**Table 4:** Test results of five alternative nested models for full path model and relative energy intake and relative REE as outcome variables.

Early Nutrition		Relative Energy Intake *		Relative Energy Expenditure *	
	Nested Model **	χ^2^ (*df*)	*p*-Value	χ^2^ (*df*)	*p*-Value
Mean energy intake (10 kcal/kg/day)	1	3.474 (3)	0.32	3.661 (3)	0.30
	2	0.387 (1)	0.53	0.813 (1)	0.37
	3	1.398 (1)	0.24	0.926 (1)	0.34
	4	3.503 (1)	0.06	0.000 (1)	1.00
	5	13.88 (1)	<0.001	5.889 (1)	0.02
Mean protein intake (g/kg/day)	1	1.371 (3)	0.71	0.942 (3)	0.82
	2	0.358 (1)	0.55	0.820 (1)	0.37
	3	1.416 (1)	0.23	1.096 (1)	0.30
	4	5.697 (1)	0.02	0.000 (1)	1.00
	5	7.521 (1)	0.006	7.938 (1)	0.004
Mean fat intake (g/kg/day)	1	3.641 (3)	0.30	3.703 (3)	0.30
	2	0.329 (1)	0.57	0.744 (1)	0.39
	3	1.050 (1)	0.31	0.702 (1)	0.40
	4	4.527 (1)	0.03	0.000 (1)	1.00
	5	10.54 (1)	0.001	6.163 (1)	0.01

(*) Full path model presented in Figure 1; (**) In these models, nutritional intake after Week 3 of life is omitted as statistically non-significant. Models adjusted for birthweight SD score, sex, age at clinical examination, gestational age, highest parental education, maternal smoking during pregnancy, maternal preeclampsia and neonatal exposures to treatment in ventilator (days), bronchopulmonary dysplasia, septicemia, exchange transfusion, or persistent ductus arteriosus. Five alternative nested models (see labels of path coefficients in Figure 1): Model 1: Several paths between body weight variables and neonatal nutrition variables are fixed as 0 (i.e., rwb22 = 0, wrb41 = 0, wrb31 = 0 and wb42 = 0). Tested against full model. Model 2: Path from weight SD score at three weeks age to outcome fixed as 0 (i.e., b5 = 0). Tested against Model 2. Model 3: Path from weight SD score at six weeks age to outcome fixed as 0 (i.e., b6 = 0). Tested against Model 3. Estimates of this model are shown in Figure 2 and Supplementary Figures S1 and S2. Model derived direct, indirect and total effects are shown in Table 5. Model 4: Path from weight SD score at nine weeks age to outcome fixed as 0 (i.e., b7 = 0). Tested against Model 4. Model 5: Path from nutritional intake from the first three weeks of life to outcome fixed as 0 (i.e., b1 = 0). Tested against Model 5.

**Table 5:** Model derived effect estimates with 95% bias corrected confidence intervals for relative energy intake in adult age.

Early Nutrition	Relative Energy Intake		
	Direct Effect: Estimate (95% CI)	Indirect Effect: Estimate (95% CI)	Total Effect: Estimate (95% CI)
Mean energy intake (10 kcal/kg/day)	−2.14 (−3.77; −0.62)	−0.37 (−1.02; 0.03)	−2.51 (−3.92; −1.06)
Mean protein intake (g/kg/day)	−5.96 (−12.44; −0.59)	−1.73 ( −4.65; −0.09)	−7.68 (−13.60; −2.12)
Mean fat intake (g/kg/day)	−2.45 (−4.81; −0.33)	−0.54 (−1.36; 0.01)	−2.98 (−5.24; −0.81)

## Supplementary Materials

The following are available online at www.mdpi.com/2072-6643/9/12/1282/s1, Figure S1: Graphical representation of path model estimated for total energy intake, body weight with four time points and relative energy intake (*n* = 109), Figure S2: Graphical representation of path model estimated for protein intake during the first three weeks of life, body weight with four time points and relative energy intake in adult age (*n* = 109). Table S1: The association between the intakes of energy and nutrients during the first three weeks of life and food and nutrient intake in adulthood, Table S2: Sensitivity analysis results for effects of protein intake and fat intake on relative energy intake.

## Appendix A

### Appendix A1. Statistical Methods—Description of the Path Analysis

Path analysis is a multivariate analysis which can be used in evaluating causal models by examining the relationships between multiple variables. All variables in path model may be explanatory variables, dependent variables or both. Path analysis is represented using a path diagram with hypothesized relationships between variables. Hypothesized full path model includes all paths which may be relevant between variables. Significance of paths may be tested by comparing nested models. A nested model excludes one or more paths included in full path model. The results of path analysis reveal the strength of direct (directly from one variable to another) and indirect (mediated by one or more variables) relationships, as well as the total effect among variables examined. In the present study, the direct effect is the part of the effect of early nutrition not mediated through available growth measures. The indirect effect is the effect mediated by the variables along the path. The total effect is the sum of these effects.

In this study five alternative sub models, as sub models for full path model represented in Figure 1, were analyzed (Table 4). As a first sub model, a best fitting model for neonatal nutrition variables was fitted, excluding nonsignificant parameters concerning relationships between body weight and neonatal nutrition. Second and third sub model exclude a direct path from weight SD score at three weeks age to dependent variable and direct path from weight SD score at six weeks to dependent variable. Fourth submodel excludes path from weight SD score at nine weeks age to dependent variable. Nonsignificant drop in chi-square for this model compared to previous model, rules out the possibility of mediation effect. Finally, direct path from neonatal nutrition during first three weeks to dependent variable was excluded. Nonsignificant drop in chi-square for this model compared to previous model rules out all effects of neonatal nutrition to outcome.

Estimation results and bias corrected 95% confidence intervals shown in tables and figures are based on third sub model that includes direct and indirect paths from neonatal nutrition during first three weeks of life to dependent variable.

All path models were adjusted for birth weight SD score, sex and age at clinical examination. Additionally, all models were adjusted for gestational age, highest parental education, maternal smoking during pregnancy, and maternal preeclampsia. Finally, neonatal exposures to treatment in ventilator (days), BPD, septicemia, exchange transfusion, and persistent ductus arteriosus were added as additional adjusting variables.

### Appendix A2. Missing Data

The missing data on two later time points of body weight data were a main concern in modeling. On a sample of relative energy intake as an outcome with 109 observations, weight SD score at six weeks age has 10 (9.2%) missing observations and weight SD score at nine weeks age has 37 (33.9%) missing observations. To evaluate the sensitivity of model derived effect estimates to missing values, especially for six weeks and nine weeks from birth, all path models were further adjusted for approximate time when a child was discharged from hospital by using two dummy variables for these time points (coded as 0 = at hospital, and 1 = discharged).

Multiple imputations were used as a sensitivity analysis for model effects that might have been dependent on missing data on birthweight SD scores [58]. Ten imputed samples were estimated by using multiple imputation of chained equation (ICE). Path model estimation on imputed datasets was then carried out using Mplus. Calculations of 95% bias corrected confidence intervals were performed by using 1000 bootstrap samples generated by Stata after which 10 imputed datasets were estimated for each of 1000 bootstrap samples and path model estimation was carried out in Mplus. Finally, bias corrected 95% confidence intervals for direct, indirect and total effects were estimated in Stata by using 1000 bootstrap estimates of indirect, direct and total effects obtained from separate Mplus path analyses.

### Appendix A3. Model Fit Indices

For evaluating model fit, we used besides chi-Square test of model fit, which should be nonsignificant, Comparative Fit Index (CFI), Tucker-Lewis Index (TLI), root mean squared error of approximation (RMSEA) and standardized root mean squared residual. CFI and TLI both range between 0 and 1 (although TLI can fall outside of the range). For model with adequate fit, we considered following cutoffs: CFI ≥ 0.95, TLI ≥ 0.95, RMSEA ≤ 0.06 and SRMR ≤ 0.08 [59].
