# Supplementary material for: Neonatal Nutrition Predicts Energy Balance in Young Adults Born Preterm at Very Low Birth Weight
Source: Nutrients. 2017 Nov 24;9(12):1282. doi: 10.3390/nu9121282 (PMC5748733; doi:10.3390/nu9121282)
Supplement: Supplementary file 1 [file nutrients-09-01282-s001.pdf]

Supplementary material

Neonatal nutrition and energy balance in young adults born preterm at very low birth weight

Hanna-Maria Matinolli, Petteri Hovi, Esko Levälahti, Nina Kaseva, Patricia P Silveira, Katri Hemiö, Anna-Liisa Järvenpää, Johan G Eriksson, Sture Andersson, Jaana Lindström, Satu Männistö, Eero Kajantie.

Supplementary material:

Table S1. The association between the intakes of energy and nutrients during the first three weeks of life and food and nutrient intake in adulthood.

Table S2. Sensitivity analysis results for effects of protein intake and fat intake on relative energy intake

Figure S1. Graphical representation of path model estimated for total energy intake, body weight with four time points and relative energy intake (n= 109)

Figure S2. Graphical representation of path model estimated for protein intake during the first three weeks of life, body weight with four time points and relative energy intake in adult age (N= 109)

Supplementary material

Table S1. The association between the intakes of energy and nutrients during the first three weeks of life and food and nutrient intake in adulthood.

	Mean **energy** intake during the first three weeks of life (10kcal/kg/d)		Mean **protein** intake during the first three weeks of life (g/kg/day)		Mean **fat** intake during the first three weeks of life (g/kg/day)		Mean **carbohydrate** intake during the first three weeks of life (g/kg/day)	
	Mean diff (95% CI)	p	Mean diff (95% CI)	p	Mean diff (95% CI)	p	Mean diff (95% CI)	p
**Energy intake** (kcal/d)								
Model 1	-19.5 (-76.8; 37.7)	0.50	9.0 (-243.6; 261.5)	0.94	-19.0 (-99.1; 61.1)	0.64	3.3 (-66.4; 73.1)	0.92
Model 2	-59.9 (-126.2; 6.4)	0.08	-73.3 (-352.1; 205.5)	0.60	-63.1 (-153.8; 27.5)	0.17	-8.9 (-83.3; 65.5)	0.81
Model 3	-72.2 (-140.1; -4.2)	0.04	-121.1 (-406.7; 164.5)	0.40	-87.9 (-181.3; 5.5)	0.06	-9.2 (-84.4; 66.0)	0.81
**Fat** (E%)								
Model 1	-0.2 (-1.0; 0.6)	0.61	-0.5 (-4.2; 3.1)	0.77	-0.3 (-1.4; 0.9)	0.63	0.1 (-0.9; 1.1)	0.80
Model 2	-0.4 (-1.3; 0.6)	0.46	-1.0(-5.1; 3.0)	0.61	-0.4 (-1.7; 0.9)	0.56	-0.1 (-1.2; 0.9)	0.82
Model 3	-0.3 (-1.4; 0.7)	0.49	-1.2 (-5.4; 2.9)	0.56	-0.3 (-1.7; 1.0)	0.64	-0.1 (-1.2; 1.0)	0.81
**Saturated fat** (E%)								
Model 1	0.0 (-0.5; 0.4)	0.94	0.5 (-1.5; 2.5)	0.63	0.0 (-0.7; 0.6)	0.91	0.2 (-0.4; 0.7)	0.55
Model 2	-0.2 (-0.7; 0.3)	0.50	-0.2 (-2.4; 2.0)	0.87	-0.2 (-0.9; 0.5)	0.59	0.0 (-0.6; 0.6)	0.95
Model 3	-0.1 (-0.6; 0.4)	0.72	0.0 (-2.2; 2.3)	0.99	-0.1 (-0.8; 0.7)	0.89	0.0 (-0.6; 0.6)	0.97
**Protein** (E%)								
Model 1	0.0 (-0.4; 0.5)	0.96	-0.5 (-2.5; 1.5)	0.64	-0.1 (-0.7; 0.5)	0.74	0.0 (-0.6; 0.5)	0.94
Model 2	-0.1 (-0.6; 0.4)	0.72	-0.9 (-3.1; 1.3)	0.41	-0.2 (-1.0; 0.5)	0.51	-0.1 (-0.7; 0.5)	0.64

Supplementary material

Model 3	-0.1 (-0.6; 0.5)	0.84	-0.6 (-2.8; 1.7)	0.60	-0.2 (-1.0; 0.5)	0.55	-0.1 (-0.7; 0.5)	0.75
**Carbohydrate** (E%)								
Model 1	0.1 (-0.7; 0.9)	0.82	0.4 (-3.2; 4.1)	0.81	0.3 (-0.8; 1.5)	0.55	0.0 (-1.0; 1.0)	0.78
Model 2	0.4 (-0.5; 1.4)	0.40	1.5 (-2.5; 5.5)	0.46	0.7 (-0.6; 2.1)	0.26	0.4 (-0.7; 1.4)	0.85
Model 3	0.5 (-0.5; 1.5)	0.30	2.0 (-2.0; 5.9)	0.32	0.7 (-0.6 ;2.0)	0.28	0.5 (-0.5; 1.6)	0.53
**Alcohol (E%)**								
Model 1	0.1 (-0.6; 0.9)	0.78	0.6 (-2.7; 3.9)	0.73	0.0 (-1.0; 1.1)	0.94	-0.1 (-1.0; 0.8)	0.97
Model 2	0.0 (-0.9; 0.9)	0.93	0.5 (-3.2; 4.2)	0.79	-0.1 (-1.3; 1.1)	0.85	-0.1 (-1.; 0.9)	0.51
Model 3	-0.1 (-1.0; 0.8)	0.83	-0.2 (-3.8; 3.5)	0.94	-0.2 (-1.4; 1.1)	0.79	-0.3 (-1.3; 0.7)	0.31
**Vegetables, fruits & berries (%)***								
Model 1	-1.3 (-13.1;12.0)	0.84	-10.0 (-39.5; 34.2)	0.60	-1.3 (-13.1; 12.0)	0.84	-3.3 (-13.4; 7.9)	0.54
Model 2	0.4 (-12.9; 15.7)	0.95	-1.6 (-36.0; 51.3)	0.94	0.4 (-12.9; 15.7)	0.95	-0.9 (-11.6; 11.2)	0.88
Model 3	0.7 (-12.5; 15.8)	0.92	6.0 (-30.2; 60.8)	0.78	0.7 (-12.5; 15.8)	0.92	1.6 (-8.9; 13.3)	0.78
**Cereals (%)***								
Model 1	-18.9 (-32.0; -3.1)	0.02	-51.0 (-77.7; 7.6)	0.08	-27.6 (-43.4; -7.4)	0.01	-11.0 (-28.5; 10.7)	0.29
Model 2	-16.1 (-32.2; 3.7)	0.10	-43.3 (-76.3; 35.5)	0.20	-25.2 (-43.8; -0.7)	0.05	-3.5 (-23.6; 21.9)	0.76
Model 3	-14.2 (-30.9; 6.7)	0.17	-36.0 (-73.7; 55.7)	0.32	-25.1 (-44.2 ;0.6)	0.06	-1.0 (-21.7; 25.0)	0.93
**Milk products (%)***								
Model 1	1.6 (-8.7; 13.0)	0.77	3.9 (-35.0; 66.0)	0.87	-1.0 (-14.7; 14.9)	0.89	8.0 (-5.1; 22.8)	0.24

Supplementary material

Model 2	-1.2 (-13.0; 12.3)	0.86	-3.5 (-42.8; 62.7)	0.89	-4.3 (-19.5; 13.7)	0.61	7.0 (-6.9; 23.0)	0.33
Model 3	3.3 (-9.1; 17.2)	0.62	18.3 (-29.3; 98.2)	0.52	0.8 (-15.2; 20.0)	0.93	9.9 (-3.9; 25.6)	0.17
**Meat (%)***								
Model 1	-3.0 (-15.3; 11.2)	0.67	-19.3 (-55.6; 46.5)	0.48	-7.6 (-23.5; 11.6)	0.41	-0.6 (-15.7; 17.2)	0.94
Model 2	-11.6 (-24.5; 3.6)	0.13	-38.1 (-67.6; 18.5)	0.15	-16.9 (-32.8; 2.8)	0.09	-6.9 (-21.7; 10.8)	0.42
Model 3	-14.4 (-26.8; 0.2)	0.05	-43.1 (-70.0; 8.0)	0.08	-19.9 (-35.3; -0.8)	0.04	-7.3 (-21.8; 9.7)	0.37
**Sugar & sweets (%)***								
Model 1	-2.0 (-18.0; 17.1)	0.82	-38.2 (-71.6; 34.4)	0.22	-6.3 (-26.9; 20.1)	0.61	6.9 (-13.8; 32.6)	0.54
Model 2	-2.4 (-21.4; 21.4)	0.83	-42.0 (-76.1; 40.5)	0.23	-7.7 (-31.2; 23.7)	0.59	9.9(-13.3; 39.2)	0.43
Model 3	2.8 (-18.0; 29.2)	0.81	-31.1 (-72.6; 73.0)	0.42	-0.7 (-27.2; 35.4)	0.96	12.4 (-11.7; 42.9)	0.34

Adjusted models were adjusted for:

Model 1: sex, age at clinical examination (and total energy intake in the analyses for food groups)

Model 2: model 1 + gestational age, birth weight SD score, duration of mechanical ventilation, bpd, sepsis, PDA, maternal smoking during pregnancy and pre-eclampsia

Model 3: model 2 + current BMI, smoking, living at parental home, parental education

*values are backtrasformed from natural logarithms and therefore presented as percentages

Supplementary material

Table S2. Sensitivity analysis results for effects of protein intake and fat intake on relative energy intake.

		Sensitivity model 1*	Sensitivity model 2**	Sensitivity model 3***
Independent variable	Effect	Estimate (95%CI)	Estimate (95%CI)	Estimate (95%CI)
Protein intake				
0-2 weeks	Indirect	-1.34 (-4.06;-0.07)	-1.49 (-4.27;-0.26)	-1.18 (-3.90;-0.25)
	Direct	-5.96 (-12.30;-0.50)	-5.96 (-11.79;-0.14)	-5.96 (-11.79;-0.14)
	Total	-7.30 (-13.28;-1.71)	-7.46 (-13.44;-1.96)	-7.14 (-13.20;-1.83)
Fat intake				
0-2 weeks	Indirect	-0.43 (-1.18;-0.01)	-0.47 (-1.30;-0.08)	-0.38 (-0.90;-0.20)
	Direct	-2.45 (-4.82;-0.31)	-2.47 (-4.61;-0.41)	-2.47 (-4.63;-0.48)
	Total	-2.88 (-5.22;-0.72)	-2.94 (-5.07;-0.91)	-2.85 (-4.73;-0.50)

*) Corresponds to model 3 in table 4 additionally adjusted for age at hospital discharge

**) Corresponds to model 3 in table 4 with multiple imputation

***) Corresponds to model 3 in table 4 additionally adjusted for age at hospital discharge, with multiple imputation

Supplementary material

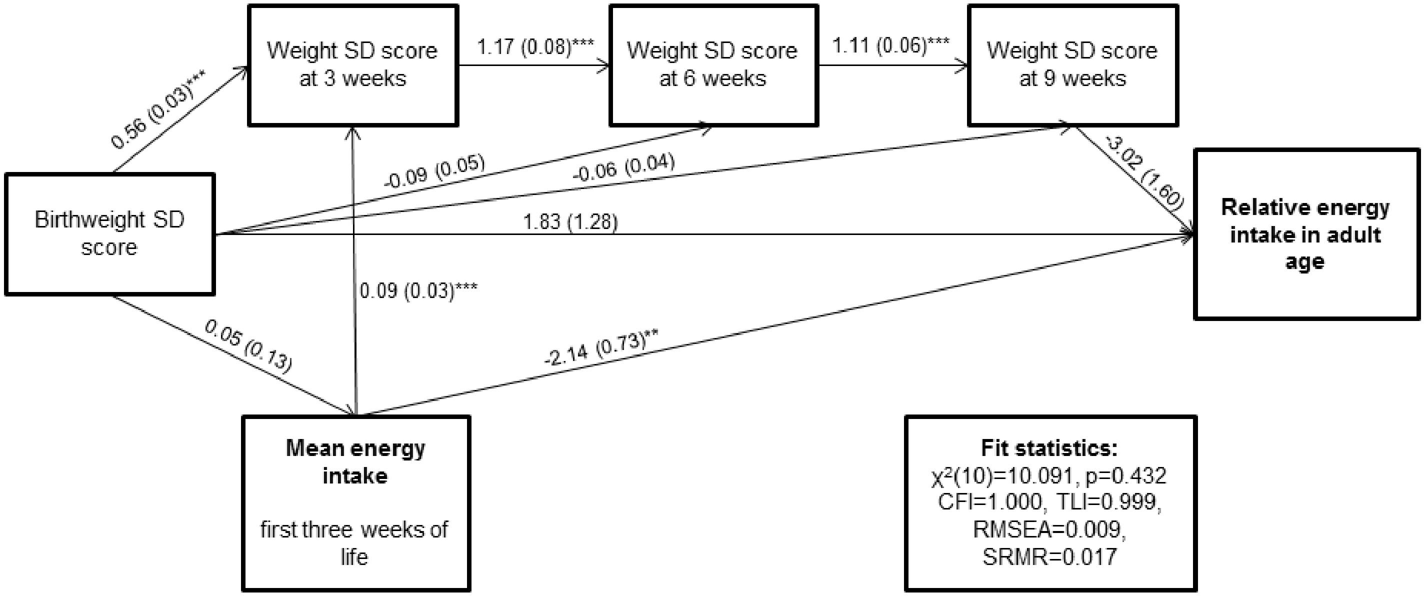

Figure S1. Graphical representation of path model estimated for total energy intake, body weight with four time points and relative energy intake (n= 109). Paths represent path coefficient and standard error in parenthesis. Significance of estimated path is noted as *) p<0.05, **) p<0.01 and ***) p<0.001 . Note that figure also includes some nonsignificant paths: most of the paths from birth weight SD score and path from weight SD score at 9 weeks to outcome.

Supplementary material

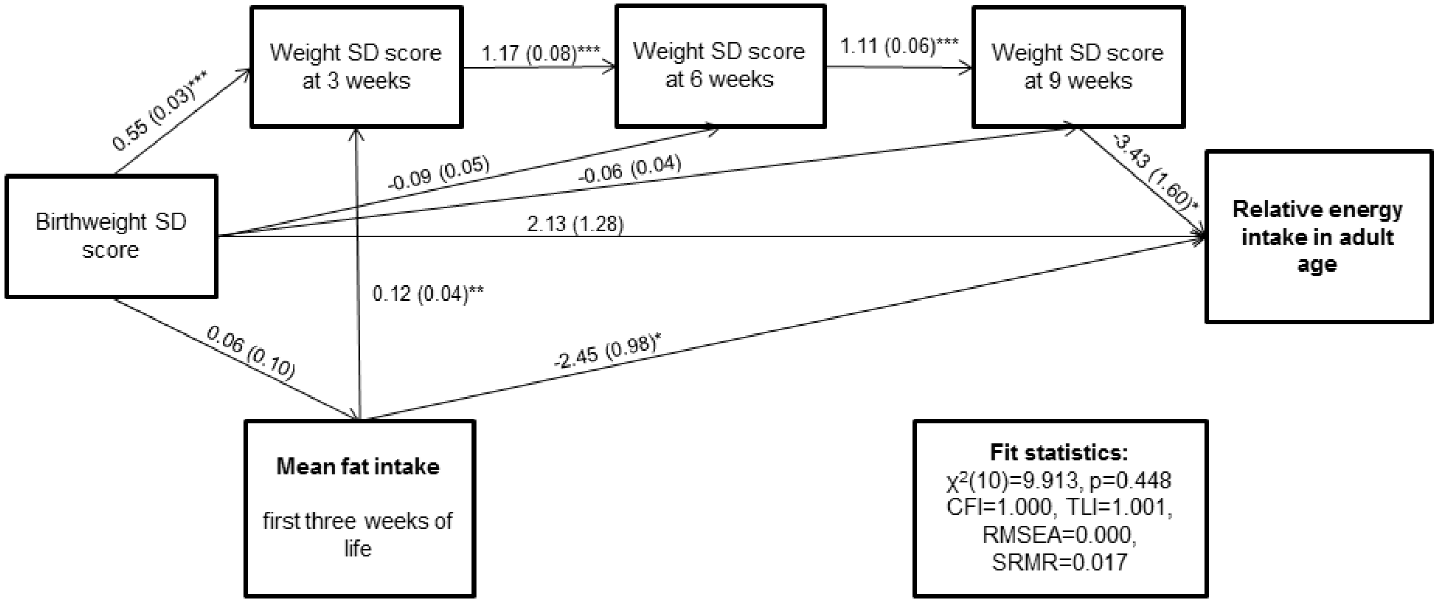

Figure S2. Graphical representation of path model estimated for protein intake during the first three weeks of life, body weight with four time points and relative energy intake in adult age (N= 109). Paths represent path coefficient and standard error in parenthesis. Significance of estimated path is noted as *) p<0.05, **) p<0.01 and ***) p<0.001 . Note that figure also includes some nonsignificant paths: most of the paths from birth weight SD score.
